# Exploring Efflux as a Mechanism of Reduced Susceptibility towards Biocides and Fluoroquinolones in *Staphylococcus pseudintermedius*

**DOI:** 10.3390/ani13071270

**Published:** 2023-04-06

**Authors:** Marta Leal, Catarina Morais, Bárbara Ramos, Constança Pomba, Patrícia Abrantes, Sofia Santos Costa, Isabel Couto

**Affiliations:** 1 Global Health and Tropical Medicine, GHTM, Instituto de Higiene e Medicina Tropical, IHMT, Universidade Nova de Lisboa, UNL, Rua da Junqueira 100, 1349-008 Lisbon, Portugal; 2 CIISA, Centre of Interdisciplinary Research in Animal Health, Faculty of Veterinary Medicine, University of Lisbon, Avenida da Universidade Técnica, 1300-477 Lisbon, Portugal; 3 GeneVet, Laboratório de Diagnóstico Molecular Veterinário, Rua Quinta da Nora Loja 3B, 2790-140 Carnaxide, Portugal

**Keywords:** *Staphylococcus pseudintermedius*, efflux, biocides, fluoroquinolones, resistance, companion animals

## Abstract

**Simple Summary:**

*Staphylococcus pseudintermedius* is the main bacterial agent of skin and soft tissue infections in companion animals. The rising antimicrobial resistance in this species is a public health concern. Efflux activity is a resistance mechanism poorly characterized for this bacterium. This study aimed to evaluate efflux as contributor of biocide and fluoroquinolone resistance in *S. pseudintermedius*. Determination and application of cut-off values detected a non-wild type population against the biocide tetraphenylphosphonium bromide, linked to increased efflux activity. Further characterization of this efflux activity demonstrated that it is strain-specific and glucose-dependent. Fluoroquinolone resistance was mainly related to target mutations, which may be masking the contribution of efflux. This study highlights the relevance of efflux-mediated resistance in *S. pseudintermedius*, particularly to biocides, and provides a methodological basis for further studies on the efflux activity on this important veterinary pathogen.

**Abstract:**

*Staphylococcus pseudintermedius* is the main bacterial cause of skin and soft tissue infections (SSTIs) in companion animals, particularly dogs. The emergence of methicillin-resistant *S. pseudintermedius* (MRSP) strains, frequently with multidrug resistance phenotypes is a public health concern. This study aimed to evaluate efflux, a resistance mechanism still poorly characterized in *S. pseudintermedius*, as a contributor to biocide and fluoroquinolone resistance. Susceptibility to the efflux pump substrates ethidium bromide (EtBr), tetraphenylphosphonium bromide (TPP) and ciprofloxacin (CIP) was evaluated by minimum inhibitory concentration (MIC) determination for 155 SSTIs-related *S. pseudintermedius* in companion animals. EtBr and TPP MIC distributions were analyzed to estimate cut-off (CO_WT_) values. The effect of the efflux inhibitors (EIs) thioridazine and verapamil was assessed upon MICs and fluorometric EtBr accumulation assays, performed with/without glucose and/or EIs. This approach detected a non-wild type population towards TPP with increased efflux, showed to be strain-specific and glucose-dependent. Resistance to fluoroquinolones was mainly linked to target gene mutations, yet a contribution of efflux on CIP resistance levels could not be ruled out. In sum, this study highlights the relevance of efflux-mediated resistance in clinical *S. pseudintermedius*, particularly to biocides, and provides a methodological basis for further studies on the efflux activity on this important pathogen of companion animals.

## 1. Introduction

*Staphylococcus pseudintermedius* is a colonizer of the skin of companion animals, particularly dogs [1,2]. However, it is also an opportunistic agent, causing several infections, mostly skin and soft tissue infections (SSTIs), accounting for up to 92% of canine pyoderma cases [3,4]. The standard procedure to treat SSTIs caused by *S. pseudintermedius* relies on topical and/or systemic therapeutics [4,5], depending on the extension and severity of the infection [6,7]. Because these are often recurrent infections, animals are subjected to multiple and prolonged antimicrobial treatments [8,9], promoting the selection and dissemination of resistant strains with severe consequences for infection management.

Similarly to *Staphylococcus aureus*, the emergence of methicillin-resistant *S. pseudintermedius* (MRSP) strains, frequently associated with multidrug resistance (MDR) phenotypes is a public health concern [10]. One approach to mitigate the rising antibiotic resistance in this pathogen include the use of biocides on topical therapy [6,7], since these are effective and show low rates of emergence of resistance [4,11,12].

In recent years, the zoonotic potential of *S. pseudintermedius* has gained interest. Although human infections caused by *S. pseudintermedius* are rare [13], they can become severe, including prosthetic infections, endocarditis and bacteremia [1,13,14]. Risk factors associated with these infections include advanced age, implants, skin and wound infections and close contact with companion animals [1,15], the latter suggesting zoonotic transmission [2]. The rising presence of companion animals in households and the inherent close contact between them and family members increase the chances of zoonotic transmission. It also increases the changes of horizontal genetic transfer of antimicrobial resistance genes between *S. pseudintermedius* and other staphylococci (or vice-versa) that cause infections in humans, namely *S. aureus* [1,16]. The exchange of genetic material between staphylococcal species may contribute to the escalation of antimicrobial resistance and the limitation of antimicrobials available for human and animal health.

Efflux is a first-line bacterial defense mechanism against antimicrobials that is now recognized to play a major role in the development of MDR phenotypes [17,18,19,20,21]. Efflux-mediated resistance has been well characterized in *S. aureus*, with more than 30 chromosomal or plasmid-encoded efflux pumps (EPs) described [18]. The most well studied is NorA, a chromosomally encoded MDR EP, which can extrude a variety of antimicrobials. Overexpression of its coding gene, *norA*, is related to fluoroquinolone resistance and decreased susceptibility to biocides (quaternary ammonium compounds, chlorhexidine, tetraphenylphosphonium bromide) and dyes such as ethidium bromide in *S. aureus* [18,22], in *S. epidermidis* [23] and more recently, in *S. pseudintermedius* [24]. Still, efflux-mediated resistance is scarcely characterized in *S. pseudintermedius*. In fact, to date, few chromosomal MDR efflux pumps have been described in this pathogen and only NorA has been partially characterized [13,24,25]. Other efflux systems, such as the plasmid-encoded MDR EPs QacA/B and Smr may also contribute to decreased susceptibility towards biocides and dyes in staphylococci [18,26]. As such, many issues remain unaddressed regarding efflux-mediated resistance in *S. pseudintermedius* and its association with antimicrobial resistance.

In a recent report on *S. pseudintermedius* causing SSTIs in companion animals in Lisbon, Portugal, we documented a high burden of antimicrobial resistance towards first and second-line antimicrobials for SSTIs management, including fluoroquinolones [27]. In that previous study, we report a high frequency of MRSP (31.0%) and MDR (45.2%) strains, as well as fluoroquinolone resistant strains [27]. In the present work, we further analyze these 155 *S. pseudintermedius* to evaluate efflux as a contributor to biocide and fluoroquinolone resistance in *S. pseudintermedius* causing SSTIs in companion animals.

## 2. Materials and Methods

### 2.1. Bacterial Strains

The study collection comprised 155 SSTIs-related *S. pseudintermedius* isolated from companion animals (dogs = 141, cats = 3, rabbit = 1) in Lisbon district, Portugal and was recently described by Morais et al. [27]. The main phenotypic characteristics of this collection is summarized in the Supplementary Data (Table S1). In the study by Morais et al., methicillin resistance was assessed by PCR amplification of *mecA* gene complemented with oxacillin susceptibility testing by disk diffusion, susceptibility to the fluoroquinolones enrofloxacin, ciprofloxacin, pradofloxacin and moxifloxacin was evaluated by disk diffusion [27,28,29], and mutations in the quinolone resistance determining region (QRDR) in target genes were screened by sequencing [27]. Strains which presented resistance to at least one antimicrobial of at least three classes of antimicrobials were considered multidrug resistant.

*S. pseudintermedius* DSM21284^T^, *S. aureus* ATCC25923^TM^ and *S. aureus* ATCC25923_EtBr [30] were used as control strains for all assays. All strains were grown in tryptic soy broth (TSB) (Oxoid^TM^, Hampshire, UK) with shaking or in tryptic soy broth agar (TSA) (Oxoid^TM^, Hampshire, UK) at 37 °C.

### 2.2. Antibiotic, Biocides and Efflux Inhibitors

Ethidium bromide (EtBr), tetraphenylphosphonium bromide (TPP), verapamil (VER) and thioridazine (TZ) were purchased from Sigma-Aldrich (St. Louis, MO, USA). Ciprofloxacin (CIP) was purchased from Fischer Scientific (New Hampshire, NH, USA). All the solutions were prepared in deionized water on the day of the experiment.

### 2.3. Determination of Minimum Inhibitory Concentrations

Minimum inhibitory concentrations (MICs) of EtBr and TPP were determined by the two-fold broth microdilution method with cation-adjusted Mueller-Hinton broth (CAMHB, Oxoid™) [31]. MICs of CIP were also determined by the two-fold broth microdilution method and evaluated according to the CLSI breakpoints [28].

Briefly, overnight cultures were resuspended in CAMHB to achieve, a cellular suspension corresponding to McFarland 0.5 and aliquoted in 96-well plates with two-fold dilutions of the compound to be tested. The MIC values correspond to the lowest concentration of antimicrobial that presented no visible growth after 18-hour incubation at 37 °C. To evaluate the effect of efflux inhibitors on the MIC values, parallel cultures were tested in media containing varying concentrations of the antimicrobial in the absence and presence of the efflux inhibitors TZ and VER at the sub-inhibitory concentrations of 12.5 mg/L and 400 mg/L, respectively. All assays were done in duplicate, and in case of doubt, in triplicate.

### 2.4. Determination of Cut-Off (CO_WT_) Values

The analysis of EtBr and TPP MICs distributions were used to estimate cut-off values (CO_WT_). The CO_WT_ values allow to differentiate wild type populations (WT, absence of acquired resistance mechanism(s) with phenotypic expression) from non-wild type populations (NWT, presence of acquired resistance mechanism(s) with phenotypic expression) [32]. The CO_WT_ corresponds to the highest MIC value presented by the WT population, and it is expressed as WT ≤ X mg/L. The NWT population corresponds to strains with MICs > CO_WT_ [32].

The Iterative Statistical Method was used to determine the CO_WT_ value applying the ECOFFinder datasheet available at http://www.eucast.org/mic_distributions_and_ecoffs/ (accessed on 15 February 2023). This method uses a nonlinear least squares regression for subsets of a log2-normal distribution of cumulative counts of MIC data to estimate the number of strains in each subset, the log2 values of the mean MIC and associated standard deviation (SD), as well as an optimal fit to the estimated MIC distribution of the WT population [33]. The log2 values of the mean MIC and SD are used to determine the cut-off value at 99% of the WT population [33].

### 2.5. Detection of Efflux Activity by Real Time Fluorometry

The real time fluorometric assay detects efflux activity by monitoring the accumulation and extrusion of EtBr, a broad substrate of efflux pumps, due to different intensities of EtBr fluorescence inside and outside the cell [17,19,20,23,30,34].

EtBr accumulation assays were performed with increasing concentrations of EtBr, in the absence/presence of 0.4% glucose (Sigma-Aldrich).

Briefly, cultures were grown overnight at 37 °C and 180 rpm in TSB and transferred to a new medium. These fresh cultures were grown until an OD_600_ of 0.6, centrifuged and washed twice with phosphate buffer saline (PBS). Solutions (0.1 mL) were prepared containing different concentrations of EtBr (0–3 mg/L) with or without glucose 0.8% and cellular suspension (final OD_600_ = 0.3).

The EtBr accumulation assays were conducted in a Rotor-gene 3000^TM^ (Corbett Research, Mortlake, Australia), in which EtBr fluorescence was read at 530/585 nm, at the end of each 60 s cycle during 60 min, at 37 °C [34].

EtBr accumulation assays were also performed in the absence/presence of EIs, TZ and VER at sub-inhibitory concentrations (12.5 mg/L and 400 mg/L, respectively). These assays were applied as described above, except for the use of a single EtBr concentration, the EtBr equilibrium concentration, which corresponds to the equilibrium of influx and efflux of EtBr [35]. Data collected in the previous assays in this study established EtBr equilibrium concentrations for the different strains, as follows: 0.25 mg/L (DSM21284^T^, TPP^WT^ and TPP^NWT^ subgroup 3) and 0.5 mg/L (TPP^NWT^ subgroup 2). The TPP^NWT^ subgroup 1 were not included in these assays.

We also determined the relative final fluorescence (RFF), a parameter that evaluates the efflux inhibitory capacity of a particular compound. This parameter is calculated through the acquired fluorescence at minute 60, in the absence and presence of EIs [35], as follows:

(1) $$\mathrm{RFF}=\frac{\left({\mathrm{Fluorescence}}_{60\;\min}\mathrm{EtBr}+\mathrm{inhibitor}\right)-\left({\mathrm{Fluorescence}}_{60\;\min}\mathrm{EtBr}\right)}{\left({\mathrm{Fluorescence}}_{60\;\min}\mathrm{EtBr}\right)}$$

### 2.6. Detection of Plasmid-Encoded Efflux Pump Genes

Internal fragments of *qacA/B* and *smr* genes were screened using total DNA solutions (1:10) previously extracted by the boiling method [27]. The control DNAs and primers are described in Supplementary Data (Table S2) [36,37,38]. The PCR reaction mixtures (25 µL) contained: 1× *Taq* buffer (NZYTech, Lisbon, Portugal), 1.75 mM MgCl2, 0.2 mM of dNTPs, 0.4 µM of each primer and 0.75 U of *Taq* II Polimerase (NZYTech, Lisbon, Portugal), and were conducted on a thermocycler Biometra Uno II or Biometra T Personal (Analytik Jena AG, Jena, Germany). The amplification conditions were as follows: DNA was denatured at 95 °C for 4 min, followed by 35 cycles of denaturation of 1 min (*qacA/B*) or 30 s (*smr*), annealing at 40 °C for 45 s (*qacA/B*) or 48 °C for 30 s (*smr*), extension at 72 °C for 1 min (*qacA/B*) or 30 s (*smr*), followed by a step of final extension at 72 °C for 5 min.

The amplification products were visualized by electrophoresis on 1% agarose gels.

### 2.7. Statistical Analysis

The possible statistical relations between the MIC_EtBr_/MIC_TPP_ ranges and methicillin resistance phenotype (MRSP and MSSP) were analyzed with SPSS program v.26 (IBM Corp., Armonk, NY, USA) for Windows. The statistical test applied was the non-parameter Mann-Whitney-Wilcoxon test. Differences were considered statistically significant when *p* < 0.05.

## 3. Results

### 3.1. Relation between Reduced Susceptibility to TPP and EtBr and Efflux Activity

#### 3.1.1. Susceptibility Profiling towards EtBr and TPP

**Figure 1:**
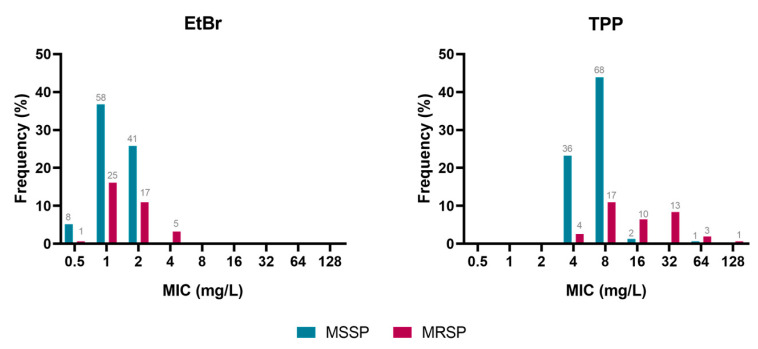
Distribution of MIC values of EtBr and TPP for the entire *S. pseudintermedius* collection (*n* = 155), according to methicillin resistance profile. Grey values indicate the number of strains. MSSP: methicillin-susceptible *S. pseudintermedius*; MRSP: methicillin-resistant *S. pseudintermedius*.

Susceptibility to EtBr and TPP was evaluated by MIC determination for all the 155 *S. pseudintermedius* clinical strains in study (Figure 1), revealing a different MIC range for each compound. For EtBr, MICs varied between 0.5–4 mg/L, while for TPP, the MICs ranged between 4–128 mg/L. Considering the profile towards methicillin, the methicillin-susceptible strains (MSSP) presented MIC_EtBr_ between 0.5–2 mg/L, while the MRSP strains displayed MIC_EtBr_ up to 4 mg/L. However, these differences were not statistically significant (*p* = 0.11).

Regarding TPP, MSSP isolates showed lower MIC values, mostly up to 8 mg/L (except for 3 strains) when compared to MRSP strains (4–128 mg/L) (Figure 1). This was a significant difference (*p* < 0.01), indicating that MRSP generally presents lower susceptibility towards TPP than MSSP strains.

#### 3.1.2. Cut-Off Values (CO_WT_) and Identification of *S. pseudintermedius* NWT Populations for EtBr and TPP

**Figure 2:**
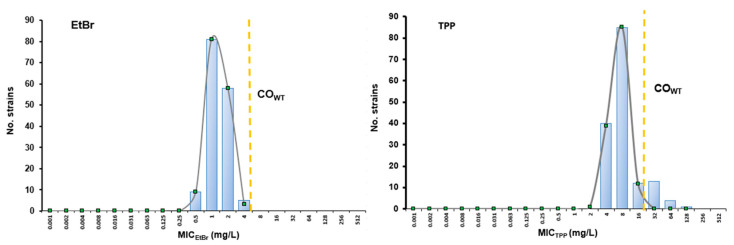
MIC distributions and cut-off (CO_WT_) values of *S. pseudintermedius* for ethidium bromide (EtBr, **left**) and tetraphenylphosphonium bromide (TPP, **right**). The CO_WT_ values were estimated by the iterative statistical method. The blue columns correspond to the MIC values determined for all strains, whereas the grey lines indicate the MIC distribution for the estimated wild type (WT) population. The dashed yellow line indicates the calculated CO_WT_ value, that corresponds to the highest MIC value for the estimated WT population (CO_WT,EtBr_ = 4 mg/L, CO_WT,TPP_ = 16 mg/L).

The CO_WT_ value corresponds to the highest MIC value presented by a population devoid of resistance mechanisms with phenotypic expression to a particular compound, named wild-type (WT) population [32]. As such, the determination of this parameter permits to distinguish microorganisms that can potentially carry resistance mechanism(s) with phenotypic expression to that compound, nominated as NWT strains [32]. These NWT strains are characterized by MIC values above the CO_WT_. This approach was used to screen for *S. pseudintermedius* strains with decreased susceptibility levels towards EtBr and/or TPP, potentially associated with efflux. The MIC_EtBr_ and MIC_TPP_ distributions of the entire collection were used to estimate CO_WT_ values by the iterative statistical method [33] using the ECOFFinder program (Figure 2). We determined CO_WT_ values of 4 mg/L and 16 mg/L for EtBr and TPP, respectively. Therefore, the NWT populations would correspond to those strains with an MIC_EtBr_ > 4 mg/L or MIC_TPP_ > 16 mg/L.

The results presented in Figure 2 and Table 1 indicate the absence of an NWT population for EtBr and the presence of an NWT population against TPP. This TPP^NWT^ population was constituted by 18 clinical strains (11.6%), 17 of which were MRSP with MDR phenotypes. This TPP^NWT^ population was characterized by significantly higher MIC_TPP_ (*p* < 0.001) and MIC_EtBr_ (*p* = 0.043) values than the TPP^WT^ population. These results indicated the presence of a mechanism of resistance towards TPP, potentially efflux, in these 18 strains.

#### 3.1.3. Assessment of Efflux Activity in S. pseudintermedius Strains

##### Effect of Efflux Inhibitors (EIs) on MIC_EtBr_ and MIC_TPP_

The effect of the EIs TZ and VER on the susceptibility levels of EtBr and TPP was evaluated in a subset of 70 clinical strains and strain DSM21284^T^ (Table 2). This subset included the 18 TPP^NWT^ strains, as well as 52 strains from the WT population (TPP^WT^). These 52 strains from the WT population, which included 22 MRSP and 30 MSSP strains, were selected according to their susceptibility towards ciprofloxacin, methicillin and other antimicrobials (multidrug resistance), previously determined [27]. We considered that efflux inhibitory activity by an EI corresponded to a reduction of the MIC_EtBr_ or MIC_TPP_ to, at least, a quarter of its original value in the presence of that EI [18,19,39].

**Figure 3:**
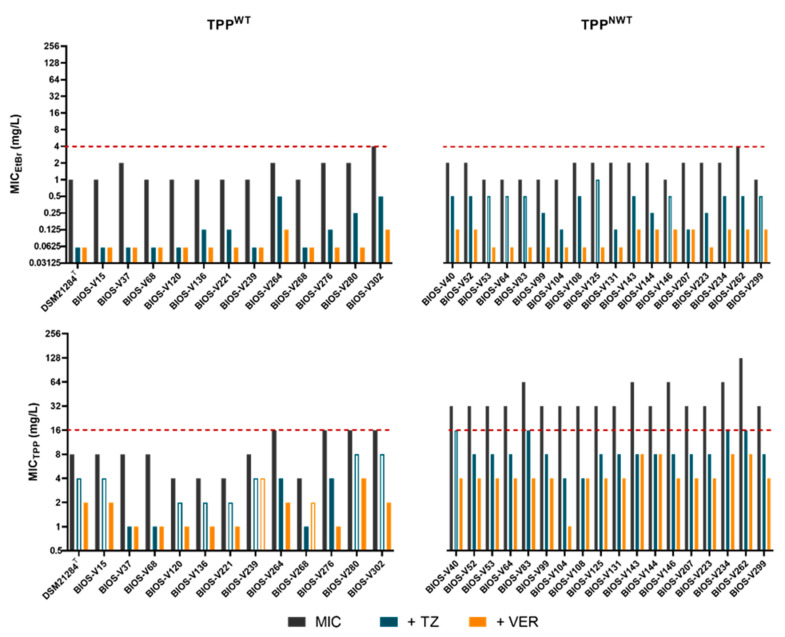
Effect of efflux inhibitors TZ and VER on MIC_EtBr_ and MIC_TPP_ in the TPP^WT^ and TPP^NWT^ population. The filled blue and orange bars represent significant reductions (≥4×) of MICs in the presence of EIs. The red dashed lines indicate the CO_WT_ value determined for each compound.

Overall, both EIs (TZ and VER), reduced the MIC_EtBr_ and MIC_TPP_ values in a scale of 2–32× of its original value (Table 2). Significant MIC_TPP_ and MIC_EtBr_ reductions (≥4-fold) were detected amongst the TPP^WT^ and TPP^NWT^ populations in the presence of the two EIs. However, this effect was particularly striking for the TPP^NWT^ population, for which the EIs reduced MIC_TPP_ to values below the *S. pseudintermedius* CO_WT_ for TPP (Figure 3). From the two EIs, VER promoted a more pronounced effect, also for a higher number of strains. These data reinforce efflux as the mechanism conferring reduced susceptibility to TPP in the TPP^NWT^ population.

##### Detection and Analysis of Efflux Activity by Real-Time Fluorometry

To confirm the increased efflux activity of the TPP^NWT^ population, the intracellular accumulation of EtBr, a broad range substrate of most bacterial MDR EPs was evaluated by real-time fluorometry.

**Figure 4:**
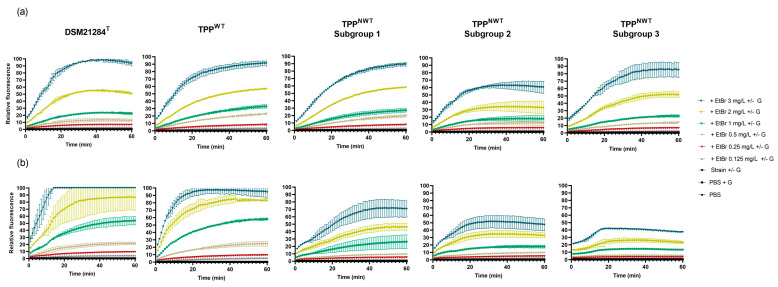
Fluorometric assays of EtBr accumulation by representative *S. pseudintermedius* strains, in (**a**) absence and (**b**) presence of 0.4% glucose (G). Data are shown for a representative strain of TPP^WT^ population (BIOS−V37) and each subgroup of TPP^NWT^ population (Subgroup 1: BIOS−V83; Subgroup 2: BIOS−V143; Subgroup 3: BIOS−V262). Data correspond to the average and standard deviation of 2–3 assays. PBS: phosphate buffer saline.

Since this methodology has been scarcely applied to *S. pseudintermedius*, it required the optimization of experimental conditions, which was carried out with strain DSM21284^T^. This strain accumulated EtBr (increasing fluorescence) proportionally to the increasing EtBr concentrations tested, reaching almost 100% of the fluorescence signal with the highest EtBr concentration tested (3 mg/L) (Figure 4a). In the presence of glucose, an energy source of efflux systems, the EtBr accumulation in this strain was slightly promoted (Figure 4b). The optimized assay was then applied to a group of representative clinical strains (*n* = 11), which included eight TPP^NWT^ and three TPP^WT^ strains. These strains were selected based on their highest MIC_TPP_ and MIC_EtBr_ values, highest inhibitory effect of EIs, as well as genotypic (clonal lineage and *agr* type) and phenotypic traits (MRSP/MSSP, resistance to fluoroquinolones) previously determined [27].

Analysis of the EtBr accumulation profile of the selected strains revealed different accumulation patterns (Figure 4). The TPP^WT^ strains BIOS-V37, BIOS-V268 and BIOS-V276 accumulated EtBr similarly to DSM21284^T^, suggesting absence of increased efflux. As for TPP^NWT^ strains, the EtBr accumulation pattern was heterogeneous, and these strains were subdivided into three subgroups (Figure 4). Subgroup 1, constituted by strains BIOS-V83, BIOS-V223 and BIOS-V146, accumulated EtBr in a manner similar to DSM21284^T^ and TPP^WT^ strains in the absence of glucose, whereas with glucose accumulated less EtBr. Subgroup 2, formed by strains BIOS-V104, BIOS-V143 and BIOS-V234, showed reduced EtBr accumulation either in the absence or presence of glucose, in comparison to DSM21284^T^ and TPP^WT^ strains. Finally, subgroup 3 (strains BIOS-V99 and BIOS-V262), showed, in the absence of glucose, an accumulation pattern similar to TPP^WT^ and TPP^NWT^ subgroup 1 strains. In the presence of glucose, EtBr accumulated at very low values for all concentrations tested.

Since lower levels of EtBr accumulation indicated higher efflux of this molecule, these results indicated that TPP^NWT^ strains, particularly those of subgroups 2 and 3, have higher efflux activity than TPP^WT^ strains and DSM21284^T^.

To further support these findings, EtBr accumulation assays were also conducted in the absence/presence of EIs for six *S. pseudintermedius* clinical strains; five TPP^NWT^ assigned to subgroup 2 or 3 and one TPP^WT^ strain, as well as DSM21284^T^. The TPP^NWT^ subgroup 1 strains were not analyzed since they presented lower efflux activity in comparison to the other subgroups. To compare the effect of the EIs TZ and VER on EtBr accumulation, we determined the relative final fluorescence (RFF) value, a parameter that reflects the inhibitory efflux capacity of each EI (Table 3). RFF values higher than 1 indicate efflux inhibition [35].

The RFF values were calculated in the absence and presence of glucose and supported the previous findings of increased efflux activity in TPP^NWT^ strains from subgroup 2 and 3—Table 3. This is reflected by the lower RFF values obtained for strains with basal efflux activity (DSM21284^T^ and TPP^WT^). On the other hand, strains with augmented efflux (TPP^NWT^) were more affected by EIs. This inhibitory effect was particularly notorious in the presence of glucose, a condition of optimal efflux activity. The two EIs presented different efflux inhibitory capacities; higher for VER on all strains except one (BIOS-V99, subgroup 3), lower for TZ, with significant inhibitory effect for a single strain, assigned to TPP^NWT^ subgroup 3 (BIOS-V262). These results confirmed the higher efflux activity of TPP^NWT^ strains, which was inhibited by EIs, particularly verapamil.

#### 3.1.4. Screening of Plasmid-Encoded Efflux Pump Genes *qacA/B* and *smr*

The genes *qacA/B* and *smr* encode, respectively, the efflux pumps QacA/B and Smr, which are involved in decreased susceptibility towards biocides, like TPP, and dyes, such as EtBr [18,19,26]. Therefore, we assessed their presence by PCR, to infer on their potential contribution towards the increased efflux activity detected in TPP^NWT^ population, as well as in the remaining strains. No strains were found to carry either *qacA*/*B* or *smr* in the entire collection (*n* = 155), which indicates that the detected efflux activity originates from other efflux systems, namely chromosomally-encoded MDR EPs.

### 3.2. Relation between Resistance to Fluoroquinolones and Efflux Activity

#### Effect of Efflux Inhibitors on Ciprofloxacin (CIP) Susceptibility Levels

Fluoroquinolones are also substrates of *S. aureus* native MDR efflux systems [17,18,19]. This observation was recently extended for a reference *S. pseudintermedius* strain [24]. Thus, we aimed to assess the contribution of efflux to fluoroquinolone resistance. A previous characterization of the antimicrobial susceptibility profiles of the study collection by disk diffusion assays revealed that 24.8% of the 155 strains (*n* = 38) were resistant or intermediate to ciprofloxacin. Susceptibility to ciprofloxacin was re-evaluated by MIC determination, which agree with the disk diffusion susceptibility profiles for all strains but one, categorized as susceptible or intermediate, by disk diffusion or MIC, respectively (Table 2) [28]. The MIC_CIP_ values ranged between 0.06 and 64 mg/L for the entire collection. The selected 70 clinical strains (Table 2) presented different susceptibility phenotypes towards ciprofloxacin, namely 31 resistant strains (MIC_CIP_ > 4 mg/L), two intermediate (MIC_CIP_ = 2 mg/L) and 37 susceptible strains (MIC_CIP_ ≤ 1 mg/L). Of these, the 18 TPP^NWT^ strains included 17 ciprofloxacin resistant strains (MIC_CIP_ of 32–64 mg/L) and a single susceptible strain (MIC_CIP_ = 0.25 mg/L). In staphylococci, resistance to fluoroquinolones is mainly mediated by mutations in the QRDR of target genes *grlA/B* and/or *gyrA/B* [42], which can hinder the assessment of the efflux contribution to phenotypes.

The EIs TZ and VER revealed a mild effect upon MIC_CIP_ values for the TPP^NWT^ strains, resulting mostly in two-fold MIC reductions (Table 2). However, the EIs effect may be masked by the presence of the double mutation S80I (GrlA)/S84L (GyrA) carried by most of these strains, which are often associated with MIC_CIP_ > 32 mg/L [43,44]. The same mild effect of EIs was observed for most TPP^WT^ strains, with only four strains showing a 4-fold MIC_CIP_ reduction. Interestingly, these strains harbored no QRDR mutations or a single mutation in GrlA (S80I) and may represent a stage when resistance results from a balance between efflux and mutation acquisition.

## 4. Discussion

*S. pseudintermedius* is the main bacterial agent of pyoderma in companion animals, particularly in dogs [3,4] and the emergence of MRSP strains, often associated with MDR phenotypes, is a public health concern [10]. This study aimed to characterize efflux activity, a resistance mechanism still poorly characterized for this species, as a contributor to biocide and fluoroquinolone resistance.

### 4.1. Contribution of Efflux to Reduced Susceptibility to Biocides, EtBr and Resistance to Fluoroquinolones in S. pseudintermedius

The study initiated with the assessment of TPP and EtBr susceptibility levels for all 155 *S. pseudintermedius* in the collection and the determination of CO_WT_ to screen for NWT populations towards for these two compounds.

For EtBr, the range of MIC values (0.5–4 mg/L) is similar to the one previously described for *S. pseudintermedius* causing different infections in companion animals, except for one isolate with an MIC_EtBr_ of 32 mg/L [45]. The range of MIC_EtBr_ values found in this study for *S. pseudintermedius* is lower when compared to the ones described for *S. aureus* or *S. epidermidis*. In *S. aureus*, MIC_EtBr_ have been reported to vary between 2–32 mg/L in human [17] or veterinary clinical isolates [46]. For *S. epidermidis*, a higher MIC_EtBr_ range (1–128 mg/L) has been described for veterinary clinical isolates [47]. For all these staphylococcal species, isolates with high MIC_EtBr_ values (>8 mg/L) were mostly associated to the presence of the EP genes *qacA/B* or *smr* genes [17,45,46,47]. The *S. pseudintermedius* CO_WT_ value for EtBr established in this study, 4 mg/L, corresponded to an absence of a NWT population against EtBr, most likely due to the absence of the *qacA/B* or *smr* genes in this collection.

A higher MIC range was observed for TPP (4–128 mg/L) and MRSP strains were more prone to present higher MIC_TPP_ values (*p <* 0.001), ie, lower susceptibility to TPP. To the best of our knowledge, this is the first proposal of a CO_WT_ of *S. pseudintermedius* for TPP (16 mg/L), which is close to that already proposed for *S. aureus*, 32 mg/L [46]. The application of the CO_WT_ for TPP identified an NWT population against this biocide. Interestingly, this population was mostly constituted by MRSP-MDR strains, from the ST71-*agr*III lineage [27,40]. The predominance of the MRSP ST71 strains in the NWT population is relevant, considering that this lineage continues to be one of the most predominant in Portugal and other European countries and is associated with a high burden of antimicrobial resistance [2,48,49,50]. As for EtBr, reduced susceptibility to TPP has also been linked to the presence of plasmid-encoded EP genes, like *qacA/B* or *smr* [46] or native MDR efflux systems, such as NorA. Since this collection was devoid of plasmid-encoded EP genes, the resistance mechanism present in the TPP^NWT^ population should be associated with an increased activity of chromosomally-encoded efflux systems.

We then proceeded to the evaluation and characterization of the *S. pseudintermedius* efflux activity by two independent methods: MIC determination in the presence of efflux inhibitors and real time fluorometry [19]. Efflux inhibitors reduced MIC_EtBr_ and MIC_TPP_ values for both TPP^WT^ and TPP^NWT^ populations. However, the EIs, particularly verapamil, had a higher impact on the MIC_TPP_ values of the TPP^NWT^ population, decreasing the MIC_TPP_ to values below the CO_WT_ established for *S. pseudintermedius*, i.e., for values corresponding to the wild type population for TPP. Therefore, these results confirm the presence of increased efflux activity in the TPP^NWT^ population and its possible association with reduced susceptibility to this biocide.

These findings were corroborated by real time fluorometry. Fluorometric assays were carried out in absence and presence of glucose, which, in strains with higher efflux leads to decreased EtBr accumulation (i.e., greater EtBr efflux). This is due to the metabolization of glucose, which energizes efflux systems [19,34]. Strains with basal efflux activity, as DSM21284^T^ and strains of the TPP^WT^ population, accumulate EtBr significantly, a trait augmented in the presence of the source of energy, an unexpected finding. On the other hand, strains with increased efflux activity, as the TPP^NWT^ population, showed lower EtBr accumulation in the presence of glucose. These overall results indicate that for *S. pseudintermedius*, efflux depends on glucose as an energy source. This glucose-dependent efflux activity was already described for other staphylococci, namely *S. aureus* and *S. epidermidis* [17,19,23,30,51]. In support of these observations, the effect of EIs on EtBr accumulation was only observed in the presence of glucose. This behavior reveals the need to energize *S. pseudintermedius* efflux systems to observe the EIs effect. This hypothesis is supported by the calculated RFF values, which are only significant in the presence of glucose, particularly for verapamil. Our data also indicate that verapamil is an effective efflux inhibitor in *S. pseudintermedius*, similarly to what has been described for *S. aureus* and *S. epidermidis* [23,52].

Despite these observations, increased efflux was not detected for all TPP^NWT^ strains tested. Lack of complete agreement between the two approaches used to evaluate efflux activity may be due to differing experimental conditions, like time of exposure to the substrate (18 hours vs. 1 hour). Another possible explanation may be different extrusion efficiency rates for EtBr and TPP by the efflux pump(s) involved, as the result of the substrate binding sites established in the pump(s) as already been postulated for the *S. aureus* NorA [53,54].

Increased efflux can lead to cross-resistance to different chemical compounds, provided they are substrates of the same EPs [55]. This response has been observed in *S. aureus*, as its MDR EPs have several substrates, including fluoroquinolones [18,56]. In particular, studies from our group have demonstrated that exposure of *S. aureus* to a biocide or EtBr results in the emergence of fluoroquinolone resistance mediated solely by efflux [20,30]. Fluoroquinolones are prescribed as second tier for the treatment of canine SSTIs and their utilization should be limited [6,57]. Nevertheless, this antibiotic class is the second most prescribed, after beta-lactams, for the treatment of infections in companion animals in European countries, including Portugal [58]. The misuse of fluoroquinolones has promoted an increase of antimicrobial resistance in Portugal and worldwide [12,50,59,60]. In *S. pseudintermedius*, resistance mechanisms against fluoroquinolones include increased efflux activity and/or mutations in QRDR of the target genes, *grlA* and *gyrA* [41,44]. A previous characterization of the collection in study had revealed a significant rate (~25%) of fluoroquinolone resistance and presence of target gene mutations. In this work, we correlated this previous susceptibility data with MIC_CIP_ values. The presence of the double mutations GrlA:Ser80Ile and GyrA:Ser84Leu, was associated with a high level of resistance (MIC_CIP_ ≥ 32 mg/L). Other QRDR double mutations patterns were detected for resistant strains with lower MIC_CIP_ values (4–8 mg/L). These data suggest that fluoroquinolone resistance in *S. pseudintermedius* is mainly due to target mutations of QRDR region of *grlA*/*gyrA* as described by Loiacono and colleagues [61]. The QRDR mutations herein reported have been already associated with fluoroquinolone resistance in *S. pseudintermedius* [43,44,62,63], and are known to convey high MIC_CIP_ values, similar to the ones found in this work [41,63,64]. This fact prevented the assessment of a potential effect of EIs upon MIC_CIP_ values. Yet, a significant EI effect was found for a few strains with a susceptible or intermediate phenotype that carried a single GrlA mutation and could represent an intermediary stage in the development of fluoroquinolone resistance. The data gathered herein for ciprofloxacin may be extrapolated for other fluoroquinolones. Descloux et. al. have reported that the presence of GrlA:Ser80Ile reduces susceptibility to enrofloxacin and Onuma et al. also reported that the presence of GrlA:Asp84Gly leads to a decreased susceptibility towards ofloxacin and enrofloxacin and resistance to levofloxacin [41,63]. Altogether, these data emphasize that the misuse of fluoroquinolones can play a role in increasing resistance in *S. pseudintermedius* and other staphylococci [20,59].

### 4.2. Misuse of TPP May Promote Emergence of Efflux-Mediated Resistance

Biocides are widely used to treat and prevent infections caused by *S. pseudintermedius*, given the increasing number of infections caused by antimicrobial resistant strains. In veterinary medicine, biocides are prescribed in combination or individual therapy, depending on the severity of the lesions [4,5].

TPP is a cationic biocide with several applications, including in medicated dressings for skin infections and for domestic surfaces disinfection. This compound acts due to its ability to prevent bacteria fixation [65,66,67] and it is structurally similar to quaternary ammonium compounds, hence having the same action mechanism of disrupting cell membranes [67,68]. When TPP was introduced, it presented an important antimicrobial activity against *S. aureus*, but later revealed to be a substrate of several staphylococcal EPs associated with high MIC_TPP_ [18,22,69,70].

The reduced susceptibility towards TPP presented by *S. pseudintermedius* could be related to incorrect use as an antiseptic, or an ineffective cleaning of surfaces and objects in contact with companion animals, which allow *S. pseudintermedius* to be exposed to sub-lethal concentrations of TPP. This exposure can promote an over-expression of MDR efflux pumps, resulting in decreased susceptibility to substrates of these pumps, such TPP and other biocides and effluxable antimicrobials like fluoroquinolones [55]. The transfer of these strains between companion animals and surfaces (or vice-versa), can contribute to their dissemination [7,71,72].

Although the decreased susceptibility to biocides used in veterinary practice may be considered not clinically relevant, since the in-use concentrations applied are much higher [16,45,73], the data obtained in this work highlight the potential decrease in susceptibility mediated by efflux and the need to use biocides prudently to prevent the spread of *S. pseudintermedius* strains with reduced susceptibility, as already described in *S. aureus* [18,19,20,74,75,76].

## 5. Conclusions

This study highlights the importance and the need of characterizing efflux in *S. pseudintermedius* as a resistance mechanism to fluoroquinolones and biocides. The use of these antimicrobial agents in veterinary therapy is put at risk by the decreased susceptibility linked to efflux, as illustrated in this work, which demonstrates that prudence is required in the use of these agents.

Surveillance of the emergence of strains with increased efflux activity, using methodological approaches such as the ones optimized and applied in this study will be crucial for the control of infections caused by this important pathogen of companion animals.

## Figures and Tables

**Table 1:** Cut-off (CO_WT_) values of *S. pseudintermedius* for EtBr and TPP. The CO_WT_ values and estimated wild-type (WT) and non-wild type (NWT) populations were determined based on MIC distributions.

	CO_WT_ 99%	SD (log_2_)	WT Population		NWT Population	
			X ≤ CO_WT_	No. Strains (%)	X > CO_WT_	No. Strains (%)
EtBr	4 mg/L	0.56	≤4 mg/L	155 (100%)	>4 mg	0 (0%)
TPP	16 mg/L	0.52	≤16 mg/L	137 (88.4%)	>16 mg/L	18 (11.6%)

SD: Standard deviation; WT: Wild-type; NWT: Non-wild type.

**Table 2:** Main characteristics of *S. pseudintermedius* strains selected for assays with EIs. Bold values indicate the MIC reduction in the presence of EI to, at least, a quarter of its original MIC value.

Category	Strain	Characteristics ^1,2^				Mutations in QRDR ^1^		MIC (mg/L)								
		MRSP MSSP	MDR	FQ	ST-*agr*	GrlA	GyrA	TPP	+TZ	+VER	EtBr	+TZ	+VER	CIP	+TZ	+VER
Type strain	DSM21284^T^	MSSP	MDR	FQ^S^	ST63-*agr*IV ^3^	-	-	8	4	**2**	1	**<0.06**	**<0.06**	0.125	0.125	0.125
TPP^NWT^	BIOS-V40	MRSP	MDR	FQ^R^	ST71-*agr*III	S80I	S84L	32	16	**4**	2	**0.5**	**0.125**	64	32	64
	BIOS-V52	MRSP	MDR	FQ^R^	ST71-*agr*III	S80I	S84L	32	**8**	**4**	2	**0.5**	**0.125**	64	32	64
	BIOS-V53	MRSP	MDR	FQ^R^	ST71-*agr*III	S80I	S84L	32	**8**	**4**	1	0.5	**<0.06**	64	64	32
	BIOS-V64	MRSP	MDR	FQ^R^	ST71-*agr*III	S80I	S84L	32	**8**	**4**	1	0.5	**<0.06**	64	64	64
	BIOS-V83	MRSP	MDR	FQ^R^	ST71-*agr*III	S80I	S84L	64	**16**	**4**	1	0.5	**<0.06**	64	32	64
	BIOS-V99	MRSP	MDR	FQ^R^	ST71-*agr*III	S80I	S84L	32	**8**	**4**	1	**0.25**	**<0.06**	64	32	64
	BIOS-V104	MRSP	MDR	FQ^R^	ST71-*agr*III	S80I	S84L	32	**4**	**1**	1	**0.125**	**<0.06**	32	32	32
	BIOS-V108	MRSP	MDR	FQ^R^	ST71-*agr*III	S80I	S84L	32	**4**	**4**	2	**0.5**	**<0.06**	32	32	32
	BIOS-V125	MRSP	MDR	FQ^R^	ST71-*agr*III	S80I	S84L	32	**8**	**4**	2	1	**<0.06**	64	32	64
	BIOS-V131	MRSP	MDR	FQ^R^	ST71-*agr*III	S80I	S84L	32	**8**	**4**	2	**0.125**	**<0.06**	64	64	64
	BIOS-V143	MRSP	MDR	FQ^R^	ST71-*agr*III	S80I	S84L	64	**8**	**8**	2	**0.5**	**0.125**	64	32	32
	BIOS-V144	MRSP	MDR	FQ^R^	ST71-*agr*III	S80I	S84L	32	**8**	**8**	2	**0.25**	**0.125**	64	32	32
	BIOS-V146	MRSP	MDR	FQ^R^	ST71-*agr*III	S80I	S84L	64	**8**	**4**	1	0.5	**0.125**	64	32	64
	BIOS-V207	MRSP	MDR	FQ^R^	ST71-*agr*III	S80I	S84L	32	**8**	**4**	2	**0.125**	**0.125**	64	64	64
	BIOS-V223	MRSP	MDR	FQ^R^	ST71-*agr*III	S80I	S84L	32	**8**	**4**	2	**0.5**	**<0.06**	64	32	64
	BIOS-V234	MSSP	No	FQ^S^	ST2194-*agr*III	-	-	64	**16**	**8**	2	**0.5**	**0.125**	0.25	0.125	0.125
	BIOS-V262	MRSP	MDR	FQ^R^	ST118-*agr*II	S80I	S84L	128	**16**	**8**	4	**0.5**	**0.125**	64	32	64
	BIOS-V299	MRSP	MDR	FQ^R^	ST71-*agr*III	S80I	S84L	32	**8**	**4**	1	0.5	**0.125**	32	---	---
TPP^WT^	BIOS-V7	MRSP	MDR	FQ^S^	ST157-*agr*IV	-	-	4	---	---	1	**0.06**	**0.03**	1	1	1
	BIOS-V10	MSSP	No	FQ^S^	ST2095-*agr*IV	-	-	8	4	4	1	**0.25**	**0.125**	0.06	0.06	0.06
	BIOS-V11	MSSP	No	FQ^S^	ST2054-*agr*III	-	-	4	4	---	1	**0.125**	**0.03**	0.06	0.06	0.06
	BIOS-V15	MSSP	No	FQ^S^	*---/agr*III	-	-	8	4	**2**	2	**0.125**	**<0.06**	0.125	0.125	0.125
	BIOS-V25	MRSP	MDR	FQ^S^	ST157-*agr*IV	-	-	4	---	---	2	**0.125**	**0.5**	1	0.5	0.5
	BIOS-V26	MRSP	No	FQ^S^	ST157-*agr*IV	-	-	8	8	4	0.5	**0.06**	**0.03**	0.25	0.125	0.125
	BIOS-V29	MRSP	No	FQ^S^	ST2055-*agr*I	-	-	8	4	4	1	**0.03**	**0.03**	0.125	0.125	0.125
	BIOS-V37	MSSP	No	FQ^S^	*---/agr*IV	-	-	8	**1**	**1**	2	**<0.06**	**<0.06**	0.25	0.125	0.125
	BIOS-V39	MSSP	MDR	FQ^S^	ST2096-*agr*III	S80I	-	8	4	4	2	**0.125**	**0.03**	1	**0.25**	0.5
	BIOS-V48	MSSP	No	FQ^S^	---/*agr*III	-	-	4	---	---	0.5	**0.125**	**0.0015**	0.25	**0.06**	**0.06**
TPP^WT^	BIOS-V65	MSSP	No	FQ^S^	---/*agr*IV	-	-	8	4	4	2	**0.125**	**0.03**	0.25	0.25	0.25
	BIOS-V68	MSSP	MDR	FQ^S^	ST2097-*agr*IV	---	---	8	**1**	**1**	1	**<0.06**	**<0.06**	0.5	0.5	0.5
	BIOS-V79	MSSP	MDR	FQ^S^	ST2098-*agr*I	---	---	8	8	---	1	**0.125**	**0.03**	0.125	0.125	0.125
	BIOS-V84	MSSP	MDR	FQ^R^	ST2099-*agr*IV	D84G	S84L	8	---	---	1	**0.06**	**0.06**	8	8	8
	BIOS-V90	MSSP	No	FQ^S^	ST2100-*agr*III	---	---	8	8	4	1	**0.125**	**0.06**	0.25	0.125	0.25
	BIOS-V96	MSSP	MDR	FQ^S^	ST2101-*agr*IV	S80R	-	4	4	2	1	**0.06**	**0.06**	1	0.5	0.5
	BIOS-V97	MRSP	MDR	FQ^R^	ST71-*agr*III	S80I	S84L	16	**4**	**4**	1	**0.25**	**0.125**	64	32	32
	BIOS-V101	MSSP	MDR	FQ^R^	ST2102-*agr*II	S80R	D83N	8	4	4	1	**0.06**	**0.06**	2	2	2
	BIOS-V103	MSSP	No	FQ^S^	---/*agr*II	S80R	-	8	8	8	1	0.5	0.5	1	0.5	0.5
	BIOS-V105	MRSP	MDR	FQ^R^	ST2056-*agr*II	S80I	S84L	8	4	4	1	**0.125**	**0.06**	64	32	32
	BIOS-V106	MSSP	No	FQ^S^	---/*agr*III	-	-	4	---	---	1	**0.25**	**0.06**	0.25	0.25	0.25
	BIOS-V116	MSSP	MDR	FQ^S^	---/*agr*IV	S80R	-	4	---	---	1	---	---	1	0.5	0.5
	BIOS-V119	MRSP	MDR	FQ^S^	ST2057-*agr*III	-	-	4	4	4	0.5	**0.06**	**0.015**	0.25	0.25	0.25
	BIOS-V120	MSSP	No	FQ^S^	ST2103-*agr*III	---	---	4	2	1	1	**<0.06**	**0.03**	0.06	0.06	0.06
	BIOS-V122	MRSP	MDR	FQ^S^	ST2104-*agr*IV	S80I	-	8	8	4	1	**0.125**	**0.06**	2	**0.5**	1
	BIOS-V127	MRSP	MDR	FQ^S^	ST157-*agr*IV	S80I	-	16	16	8	2	**0.25**	**0.03**	1	1	1
	BIOS-V136	MSSP	MDR	FQ^S^	ST2058-*agr*III	-	-	4	2	**1**	1	**0.125**	**0.03**	0.25	0.125	0.25
	BIOS-V164	MSSP	MDR	FQ^S^	ST1350-*agr*IV	-	-	16	8	8	1	**0.125**	**0.06**	0.125	0.06	0.06
	BIOS-V175	MSSP	MDR	FQ^S^	ST555-*agr*IV	---	---	4	2	---	1	**0.06**	**0.03**	0.06	0.06	0.06
	BIOS-V176	MSSP	No	FQ^S^	---/*agr*II	S80I	-	8	---	---	2	**0.5**	**0.125**	1	0.5	0.5
	BIOS-V179	MRSP	MDR	FQ^R^	ST2059-*agr*III	D84N	S84L	8	8	8	1	**0.06**	**0.125**	4	2	4
	BIOS-V190	MSSP	MDR	FQ^S^	ST2106-*agr*I	---	---	8	8	4	0.5	**0.03**	**0.03**	0.06	0.06	0.06
	BIOS-V211	MSSP	No	FQ^S^	ST2107-*agr*IV	---	---	8	---	---	1	**0.25**	**0.06**	0.25	0.125	0.25
	BIOS-V212	MSSP	No	FQ^S^	ST1183-*agr*IV	S80I	-	8	8	4	2	**0.25**	**0.06**	0.125	0.06	0.06
	BIOS-V213	MRSP	MDR	FQ^R^	ST422-*agr*III	S80I	S84L	8	---	---	1	**0.125**	**0.125**	64	32	64
	BIOS-V217	MRSP	MDR	FQ^S^	ST2060-*agr*IV	-	-	8	4	4	1	**0.06**	**0.03**	0.25	0.125	0.125
	BIOS-V218	MSSP	MDR	FQ^S^	ST241-*agr*III	-	-	8	8	8	1	**0.25**	**0.125**	0.25	**0.06**	0.25
	BIOS-V224	MRSP	MDR	FQ^R^	ST45-*agr*II	S80I	S84L	8	4	**2**	1	**0.125**	**0.125**	64	64	64
	BIOS-V225	MSSP	No	FQ^S^	ST455-*agr*III	-	-	8	4	4	1	0.5	**0.125**	0.125	0.125	0.125
	BIOS-V227	MRSP	MDR	FQ^R^	ST551-*agr*III	S80I	S84L	8	4	4	1	**0.125**	**0.06**	32	32	32
	BIOS-V228	MRSP	MDR	FQ^R^	ST45-*agr*II	S80I	S84L	8	---	---	1	**0.125**	**0.125**	64	32	32
	BIOS-V230	MSSP	No	FQ^S^	---/*agr*IV	---	---	8	4	4	1	**0.06**	**0.03**	0.06	0.06	0.06
	BIOS-V231	MSSP	No	FQ^S^	---/*agr*IV	-	-	8	8	4	2	**0.25**	**0.125**	0.125	0.06	0.125
	BIOS-V240	MRSP	MDR	FQ^R^	ST2061-*agr*III	S80I	S84L	8	8	8	4	**1**	**0.125**	64	32	64
	BIOS-V242	MSSP	MDR	FQ^S^	ST2108-*agr*II	S80I	-	8	4	4	2	**0.06**	**0.03**	1	1	0.5
	BIOS-V264	MRSP	MDR	FQ^R^	ST71-*agr*III	S80I	S84L	16	**4**	**2**	4	**1**	**0.25**	64	32	64
TPP^WT^	BIOS-V268	MSSP	MDR	FQ^S^	---/*agr*II	-	-	4	**1**	2	1	**<0.06**	**<0.06**	0.25	0.125	0.125
	BIOS-V270	MRSP	MDR	FQ^R^	ST25-*agr*III	S80I	S84L	8	4	**2**	1	**0.25**	**0.125**	32	16	32
	BIOS-V276	MRSP	MDR	FQ^S^	ST497-*agr*III	D84G	-	16	**4**	**1**	2	**0.125**	**<0.06**	1	0.5	0.5
	BIOS-V280	MRSP	MDR	FQ^R^	ST71-*agr*III	S80I	S84L	16	8	**4**	4	**0.25**	**<0.06**	64	32	64
	BIOS-V292	MRSP	MDR	FQ^R^	ST45-*agr*II	S80I	S84L	8	4	4	1	**0.125**	**0.125**	32	32	32
	BIOS-V302	MRSP	MDR	FQ^R^	ST71-*agr*III	S80I	S84L	16	8	**2**	4	**0.5**	**0.125**	64	32	64

MSSP: Methicillin-susceptible *S. pseudintermedius*; MRSP: Methicillin-resistant *S. pseudintermedius*; MDR: Multidrug resistant; TPP: tetraphenylphosphonium bromide; EtBr: Ethidium bromide; CIP: Ciprofloxacin; TZ: Thioridazine; VER: Verapamil; WT: Wild type; NWT: Non-wild type; FQ^S^: Fluoroquinolone susceptible; FQ^R^: Fluoroquinolone resistant; QRDR: Quinolone resistance determining region; -: No mutation; D: Aspartate; G: Glycine; S: Serine; I: Isoleucine; L: Leucine; N: Asparagine; R: Arginine; ---: Not determined. ^1^ The main phenotypic and genotypic characteristics of the collection were previously described [27], as summarized in Section 2 and Supplementary Table S1; ^2^ Ref. [40]; ^3^ Ref. [41].

**Table 3:** RFF values for DSM21284^T^, TPP^WT^ and TPP^NWT^ strains, determined from EtBr accumulation assays in the presence of efflux inhibitors. The RFF values are presented as the average ± standard deviation of independent assays. Bold values highlight RFF values superior to 1, which indicate efflux inhibition.

Category	Strain	Relative Final Fluorescence (RFF)			
		−Glucose +TZ	−Glucose +VER	+Glucose +TZ	+Glucose +VER
Type strain	DSM21284^T^	−0.24 ± 0.00	0.54 ± 0.03	0.61 ± 0.11	**1.05** ± 0.11
TPP^WT^	BIOS-V37	−0.37 ± 0.02	−0.09 ± 0.14	0.65 ± 0.16	**1.18** ± 0.14
TPP^NWT^ Subgroup 2	BIOS-V104	0.00 ± 0.08	**1.12** ± 0.41	0.93 ± 0.16	**1.81** ± 0.17
	BIOS-V143	−0.13 ± 0.05	0.12 ± 0.05	0.82 ± 0.40	**1.36** ± 0.68
	BIOS-V234	−0.38 ± 0.11	−0.23 ± 0.04	0.89 ± 0.14	**1.59** ± 0.03
TPP^NWT^ Subgroup 3	BIOS-V99	−0.18 ± 0.13	0.63 ± 0.20	0.30 ± 0.09	0.76 ± 0.33
	BIOS-V262	−0.40 ± 0.11	0.77 ± 0.66	**1.80** ± 0.30	**2.07** ± 0.04

WT: Wild type; NWT: Non-wild type; TZ: Thioridazine; VER: Verapamil.

## Data Availability

All relevant data have been provided in the paper. Raw data can also be provided by the authors upon reasonable request.

## Supplementary Materials

The following supporting information can be downloaded at: https://www.mdpi.com/article/10.3390/ani13071270/s1, Table S1: Distribution of MRSP, MSSP and MDR phenotypes among the *S. pseudintermedius* study collection (*n* = 155); Table S2: Control strains used for the detection of plasmid-encoded efflux pump genes; Table S3: Primers used in this study.
