# Supplementary material for: Exploring Efflux as a Mechanism of Reduced Susceptibility towards Biocides and Fluoroquinolones in Staphylococcus pseudintermedius
Source: Animals (Basel). 2023 Apr 6;13(7):1270. doi: 10.3390/ani13071270 (PMC10093712; doi:10.3390/ani13071270)
Supplement: Supplementary file 1 [file animals-13-01270-s001.zip › animals-2283573-supplementary.pdf]

animals

MDPI

Article

Exploring Efflux as a Mechanism of Reduced Susceptibility towards Biocides and Fluoroquinolones in *Staphylococcus pseudintermedius*

Marta Leal[1], Catarina Morais[1], Bárbara Ramos[1], Constança Pomba[2,3], Patrícia Abrantes[1], Sofia Santos Costa[1] and Isabel Couto[1*]

[1] Global Health and Tropical Medicine, GHTM, Instituto de Higiene e Medicina Tropical, IHMT, Universidade Nova de Lisboa, UNL, Rua da Junqueira 100, 1349-008 Lisbon, Portugal
[2] CIISA, Centre of Interdisciplinary Research in Animal Health, Faculty of Veterinary Medicine, University of Lisbon, Avenida da Universidade Técnica, 1300-477, Lisbon, Portugal
[3] GeneVet, Laboratório de Diagnóstico Molecular Veterinário, Rua Quinta da Nora Loja 3B, 2790-140 Carnaxide, Portugal
* Correspondence: icouto@ihmt.unl.pt; Tel.: +351-21-3652652; Fax: +351-21-3632105

Table S1. Distribution of MRSP, MSSP and MDR phenotypes among the *S. pseudintermedius* study collection (n=155).

S. pseudintermedius clinical strains	Number of strains (n/N) (%)	Reference
MRSP	48/155 (31.0%)	[1]
MRSP non-MDR	2/48 (4.2%)	
MRSP-MDR	46/48 (95.8%)	
MSSP	107/155 (69.0%)	
MSSP non-MDR	83/107 (77.6%)	
MSSP-MDR	24/107 (22.4%)	

MRSP: Methicillin-resistant *S. pseudintermedius*; MSSP: Methicillin-susceptible *S. pseudintermedius*; MDR: Multidrug resistant. Susceptibility profiles were interpreted according to guidelines VET01S (CLSI, 2020) (clindamycin, tetracycline, enrofloxacin, pradofloxacin) [2], M100-ED32 (CLSI, 2022) (penicillin, oxacillin, gentamicin, erythromycin, trimethoprim-sulfamethoxazole, chloramphenicol, rifampicin, ciprofloxacin, moxifloxacin) [3].

Table S2. Control strains used for the detection of plasmid-encoded efflux pump genes.

Target gene	Control strain	Reference
qacA/B	*S. aureus* SM39	[4]
smr	*S. aureus* SM52	[4,5]

Table S3. Primers used in this study.

Target gene	Primers	Nucleotide Sequence (5′-3′)	Amplicon size (bp)	Reference
Detection of plasmid-encoded efflux pump genes				
qacA/B	qacA/B_FW	GCTGCATTTATGACAATGTTT	628	[6]
	qacA/B_RV	AATCCCACCTACTAAAGCAG		
smr	smr_FW	ATAAGTACTGAAGTTATTGGA AGT	285	[7]
	smr_RV	TTCCGAAAATGTTTAACGAAA CTA		
	gyrB_RV	GGATGCGGTTTTACCAGATA		

bp: Base pair; FW: Forward; RV: Reverse.

References

1. Morais, C.; Costa S.S.; Leal, M.; Ramos, B.; Andrade, M.; Ferreira, C.; Abrantes, P.; Pomba, C.; Couto, I. Genetic diversity and antimicrobial resistance profiles of *Staphylococcus pseudintermedius* associated to skin and soft-tissue infections in companion animals in Lisbon, Portugal. *Front Microbiol*, **2023**. *In press,* doi: 10.3389/fmicb.2023.1167834
2. Clinical and Laboratory Standards Institute (VET01S CLSI). Performance standards for antimicrobial disk and dilution susceptibility tests for bacteria isolated from animals. *CLSI* supplement VET01S, , 5thed. Clinical and Laboratory Standards Institute,USA, **2020**.
3. Clinical and Laboratory Standards Institute (CLSI). Performance standards for antimicrobial susceptibility testing. CLSI supplement M100, 32nd ed. Clinical and Laboratory Standards Institute, USA, **2022**.
4. Costa S.S.; Palma C.; Kadlec K.; Fessler A.T.; Viveiros M.; Melo-Cristino J.; Schwarz S.; Couto I. Plasmid-borne antimicrobial resistance of *Staphylococcus aureus* isolated in a hospital in Lisbon, Portugal. *Microb Drug Resist*, **2016**, *22*, 1-10. doi: 10.1089/mdr.2015.0352
5. Costa S.S.; Junqueira E.; Palma C.; Viveiros M.; Melo-Cristino J.; Amaral L.; Couto I. Resistance to antimicrobials mediated by efflux pumps in *Staphylococcus aureus*. *Antibiotics*, **2013**, *2*, 83–99. doi: 10.3390/antibiotics2010083
6. Anthonisen, I. L.; Sunde, M.; Steinum, T. M.; Sidhu, M. S.; Sorum, H. Organization of the antiseptic resistance gene *qacA* and Tn552-related-lactamase genes in multidrug- resistant *Staphylococcus haemolyticus* strains of animal and human origins. *Antimicrob Agents Chemother*, **2002**, *46*, 3606–3612. doi: 10.1128/AAC.46.11.3606-3612.2002
7. Bjorland, J.; Sunde, M.; Waage, S. Plasmid-borne *smr* gene causes resistance to quaternary ammonium compounds in bovine *Staphylococcus aureus*. *J Clin Microbiol*, **2001**, *39*, 3999-4004.
